# A cloud-edge-end collaborative intelligent caching method based on incremental federated learning algorithms

**DOI:** 10.1371/journal.pone.0348359

**Published:** 2026-06-03

**Authors:** Xiang Huang, Lei Jin, Kequan Lin, Wenpeng Wu, Zhenjie Lin

**Affiliations:** 1 China Southern Power Grid Company Limited, Guangzhou, China; 2 Southern Power Grid Digital Grid Research Institute Co., Ltd, Guangzhou, China; 3 China Energy Engineering Group Guangdong Electric Power Design Institute Co. Ltd., Guangzhou, China; 4 China Power Engineering Consulting Group Limited, Beijing, China; 5 Guangdong Provincial Key Laboratory of Digital Grid Technology, Guangzhou, China; Alma Mater Studiorum Universita di Bologna: Universita degli Studi di Bologna, ITALY

## Abstract

In a cloud-edge-end collaborative system, data generated by terminal devices often contains users’ sensitive information and is constantly generated and changing, leading to potential data privacy leaks in caches. Additionally, due to the inability to promptly capture these dynamic changes and the failure to consider the actual capabilities of nodes, caching strategies become outdated, resulting in reduced cache hit rates and cache imbalance issues. Therefore, this study proposes a cloud-edge-end collaborative intelligent caching method based on an incremental federated learning algorithm. First, the federated learning algorithm is used to aggregate data from terminal devices to the cloud, enabling collaborative data processing while protecting data privacy. Second, incremental learning methods are employed to continuously update terminal data, with the updated data aggregated to the cloud, thereby enabling real-time tracking of data trends and allowing cache strategies to rapidly adapt to dynamic changes in terminal data. Finally, considering the actual capabilities of nodes, the popularity of aggregated data and the weights of edge and terminal nodes are calculated. Data is cached in edge and terminal nodes in descending order of popularity and weight. When cache space is insufficient, data replacement is performed based on the importance of data within nodes, thereby completing intelligent data caching. Experimental results demonstrate that this method achieves good performance in data update aggregation, with high data caching balance and cache hit rates.

## 1. Introduction

Data generated by edge devices often contain rich information and value, playing a significant role in driving the development of smart manufacturing, smart cities, and telemedicine. However, traditional cloud computing models struggle to meet the demands of low latency, high bandwidth, and real-time processing when faced with such massive data volumes. To address this challenge, the cloud-edge-end collaborative system has emerged. This architecture decentralizes computational tasks and data storage to the network edge. It also leverages the capabilities of edge servers. As a result, it reduces the burden on cloud centers. Data can then be processed locally and responded to quickly. In the cloud-edge-end collaborative system, intelligent caching serves as a critical technology connecting the cloud, edge, and end, playing an indispensable role [1,2]. Intelligent caching not only improves data access speed and reduces network bandwidth consumption but also dynamically adjusts cache content based on user request patterns and data access frequency, achieving efficient utilization of cache resources.

However, in actual cloud edge collaborative environments, achieving efficient, secure, and adaptive intelligent caching still faces many challenges: the data generated by terminal devices often contains sensitive user information, which poses a risk of privacy leakage; The data is constantly changing dynamically, and traditional caching strategies are difficult to track and adapt in real time; In addition, node heterogeneity and resource constraints can easily lead to issues such as uneven cache allocation and decreased hit rates. Existing research often focuses on optimizing a single aspect or fails to fully consider the balance between data dynamics and privacy protection, resulting in limited effectiveness in practical deployment. To address the aforementioned issues, this paper proposes a cloud edge collaborative intelligent caching method based on incremental federated learning algorithm. This method first achieves secure aggregation of terminal data through federated learning, and completes collaborative processing while protecting data privacy; Furthermore, an incremental learning mechanism is introduced to continuously track the trend of data changes, enabling the caching strategy to dynamically adapt to terminal data updates; Finally, taking into account the popularity of data and the actual capabilities of nodes, a differentiated cache placement and replacement strategy is designed to achieve balanced allocation and efficient utilization of system cache resources. The method proposed in this article aims to improve cache hit rate and overall system performance while ensuring data privacy and real-time performance, providing a feasible solution for intelligent caching in cloud edge collaborative environments.

## 2. Related work

In recent years, researchers have proposed various methods from multiple perspectives such as content prediction, resource allocation, and privacy protection to address the challenges of intelligent caching in cloud edge collaborative environments. However, there are still several limitations. For example, the method in [3] first establishes a content popularity prediction algorithm, which uses variational autoencoders to predict users’ future content preferences based on their previous requests. Then, it completes information caching through an online algorithm based on dynamic cache content replacement. Finally, it uses a cooperative caching algorithm to improve the efficiency of information caching. However, this method does not consider the actual capabilities of nodes, resulting in high cache imbalance issues.

The method in [4] first addresses a practical scenario in collaborative caching where user devices move at different speeds, and obtains the contact duration and mutual contact time between user devices in this scenario. Second, it transforms the caching placement problem into a problem of maximizing the saved delay under capacity and deadline constraints. Finally, it uses deep reinforcement learning algorithms to solve the above problem and obtain the optimal caching scheme. However, this method ignores the fact that data is constantly generated and changing, leading to lagging caching strategies and reduced cache hit rates.

The method in [5] first conducts a detailed analysis of the impact of NUMA interleaving on the performance of a hierarchical memory system to establish the relationship between NUMA interleaving and different memory bandwidth programs. Then, it constructs a dynamic cache management scheme (T-CAT), which divides the final-level cache between near memory and far memory to mitigate performance degradation by accessing far memory, thereby achieving efficient data caching. However, this method does not consider that data often contains users’ sensitive information and the limitations of memory caching, resulting in poor data caching performance.

The method proposed in [6] introduces a priority-based deep reinforcement learning collaborative cache management method (PDRL-CM). PDRL-CM first designs a lightweight cache admission strategy that leverages the inherent and combined attributes of data. Then, it uses Monte Carlo sampling and maximum value search strategies combined with a feedforward neural network to make cache admission decisions. Second, the method treats minimizing system delay and reducing energy consumption as a joint optimization problem, using an improved deep reinforcement learning algorithm to solve this problem and making cache sharding decisions to improve cache hit rates. However, this method cannot promptly capture dynamic data changes, leading to poor cache performance.

Reference [7] proposed a robust collaborative caching framework called RoCoCache, which combines federated deep learning with active caching strategies to address challenges such as inefficient collaboration, improper resource allocation, and adversarial attacks in multi edge caching. This method first designs a partition mechanism for multidimensional cache space to achieve accurate content recommendation within the user classification interval; Secondly, a Discrete Category Variational Autoencoder (DC-VAE) was developed to improve the accuracy of content popularity prediction; Finally, a training mode based on robust federated deep learning and an active cache replacement strategy were constructed, which introduced residual based adversarial update detection and similarity based federated aggregation mechanism to resist the impact of adversarial attacks on the model, thereby optimizing cache resource allocation and improving overall cache performance. However, its federated learning mechanism is mainly aimed at static or periodically updated data scenarios, which makes it difficult to adapt to the continuous dynamic changes of terminal data in real time, resulting in lagging cache policy updates.

The method proposed in reference [8] is a lightweight traffic aware layered framework called THOAS, which supports adaptive slicing in the Space Ground Air Integrated Network (SAGIN). This method first divides SAGIN into two hierarchical structures: communication access platform and computing processing platform; Furthermore, a traffic prediction method based on self attention mechanism was designed to accurately capture the dynamic changes of traffic on various platforms and achieve resource adjustment for adaptive slicing; Finally, an improved deep reinforcement learning method with dynamic confidence intervals was developed, and knowledge distillation techniques were used to compress strategies into lightweight networks to enhance adaptability and decision-making efficiency in resource constrained SAGIN environments. However, the framework did not introduce a cross node collaborative caching mechanism, and the storage resources of edge nodes were not utilized differentially based on content popularity and node capabilities, which may result in cache redundancy or uneven utilization.

Most existing federated learning methods are targeted at static datasets. In dynamic environments, especially when the amount of data is constantly increasing, these methods may not be able to cope effectively. Therefore, to solve the problems existing in the above algorithms, this paper proposes a research on cloud-edge-end collaborative intelligent caching method based on incremental federated learning algorithm.

## 3. Terminal data aggregation and collaborative update based on incremental federated learning algorithm

The cloud-edge-end collaborative system is divided into the cloud, edge, and end nodes. The edge node aggregates and updates the data collected from various end nodes and transmits it to the cloud. The cloud then aggregates and updates the data from each edge node to achieve collaborative updates between the cloud and edge nodes. Finally, the cloud caches the data in different edge nodes based on the data characteristics and end-user requirements. Therefore, before implementing intelligent caching of cloud-edge-end data, federated learning algorithms are first used to aggregate data from various terminal devices, enabling collaborative data processing while protecting data privacy. Then, incremental methods are employed to achieve real-time data updates, enabling the caching strategy to quickly adapt to dynamic changes in terminal data.

### 3.1. Terminal data aggregation based on federated learning algorithms

**Fig 1:**
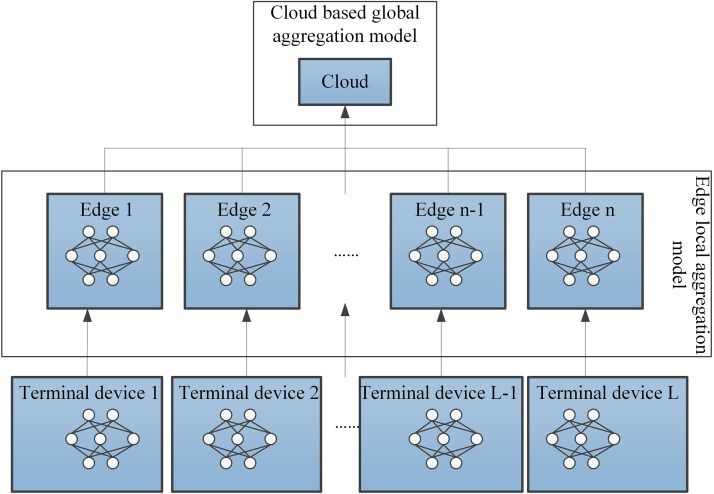
Cloud-Edge-End Collaborative Architecture Based on Federated Learning.

Federated learning algorithms primarily enable efficient machine learning across multiple terminals while ensuring the security of terminal and user data, thereby achieving data aggregation of terminal devices in a cloud-edge-end collaborative system. The specific process of aggregating terminal data in a cloud-edge-end collaborative system using federated learning algorithms is shown in Fig 1^:^

In this study, each terminal device employs a three-layer fully connected neural network as the local aggregation model, which consists of an input layer, two hidden layers (with 64 and 32 nodes respectively), and an output layer (for popularity prediction), using the ReLU activation function. This model structure balances expressiveness and computational efficiency, making it suitable for deployment on edge devices. Assume that the cloud-edge-end collaborative system contains a total of L terminals, where the L terminals are participants in the federated learning algorithm. For the data set $F_{l}$ in the terminal l(l=1,2,⋯,L), it can be represented as $F_{l}=\left\{\overset{l}{\underset{i}{c}},\overset{l}{\underset{i}{u}}\right\}$, where $\overset{l}{\underset{i}{c}}$ and $\overset{l}{\underset{i}{u}}$ represent the features and corresponding labels of the i th data point in the data set $F_{l}$, respectively.

Define $q_{l}$ as the size of the dataset in the l terminal of the cloud-edge-end collaborative system. Let ξ denote the parameters of the global model for terminal data aggregation. Then, the loss obtained by predicting the data sample $\left(c_{i},u_{i}\right)$ using ξ can be expressed as $\sigma (c_{i},u_{i};\xi )$. Before performing aggregation and training on the entire terminal dataset, all terminals must establish the same data aggregation target. The specific description of the aggregation target is as follows:


$$\underset{\xi \in R^{f}}{min}\;\sigma (c,u;\xi )=\underset{\xi \in R^{f}}{min}\frac{\text{1}}{q}\sum_{i=1}^{q}\sigma (c_{i},u_{i};\xi )$$ (1)

Where, q is the size of the dataset established based on the terminal datasets of all terminals participating in the federated learning algorithm; f is the relevant parameter.

When conducting joint training on the L terminal devices in the cloud-edge-end collaborative system, the dataset q established based on the terminal datasets participating in the federated learning algorithm can be expressed as:


$$q=\sum_{l=1}^{L}\left|F_{l}\right|=\sum_{l=1}^{L}q_{l}$$ (2)

Based on the above description, the data aggregation objectives for each terminal in formula (1) can be transformed as follows:


$$\left\{ \begin{array}{c} @l@\sigma (c,u;\xi )=\sum_{l=1}^{L}\frac{q_{l}}{q}Z_{l}(c_{l},u_{l};\xi )\\ Z_{l}(c_{l},u_{l};\xi )=\frac{q}{q_{l}}\sum_{i\in F_{i}}\sigma (c_{i},u_{i};\xi ) \end{array}\right.$$ (3)

The cloud-edge-end collaborative system first performs local aggregation of all terminal data using the local aggregation model [9] parameters, and in t=1,2,⋯ rounds of communication, the L terminal devices participating in federated learning can obtain the parameters of the global model aggregated in each round from the cloud. Assuming that ι represents the learning rate of each terminal, ι is a fixed value, and $\xi_{t}$ represents the terminal data model parameters at this moment, based on $\xi_{t}$, the average loss gradient $g_{l}$ on a single terminal dataset can be obtained. The description of $g_{l}$ is as follows:


$$g_{l}=\nabla Z_{l}(c_{l},u_{l};\xi_{t})$$ (4)

The L terminals participating in federated learning synchronously update their local aggregated model parameters and send the updated model parameters to the cloud end of the cloud-edge-end collaborative system. The cloud employs the Federated Averaging (FedAvg) algorithm to perform weighted aggregation of model parameters uploaded by each terminal, continuously optimizing the global model. Although more complex aggregation methods exist, such as FedProx or SCAFFOLD. However, the incremental learning mechanism in this system can already mitigate data distribution shifts. In addition, device heterogeneity in the experimental scenario remains relatively controllable. Therefore, FedAvg significantly reduces communication and computational overhead while ensuring aggregation effectiveness. As a result, FedAvg is more suitable for resource-constrained cloud-edge-end collaborative systems. The cloud end performs aggregation processing on the local aggregated model parameters of all terminals in each round, continuously optimizing the global aggregated model of the cloud-edge-end collaborative system. The optimization process for the global aggregated model parameters $\xi_{t+1}$ of the t+1 th round is as follows:


$$\left\{ \begin{array}{c} @l@\xi_{t+1}\leftarrow \xi_{t}-\iota \nabla \sigma (c,u;\xi )\\ \xi_{t+1}\leftarrow \xi_{t}-\iota \sum_{l=1}^{L}\frac{q_{l}}{q}\nabla Z_{l}(c_{l},u_{l};\xi_{t})\\ \xi_{t+1}\leftarrow \xi_{t}-\iota \sum_{l=1}^{L}\frac{q_{l}}{q}g_{l} \end{array}\right.$$ (5)

Since the locally aggregated model parameters for a single terminal l are $\overset{l}{\underset{t+1}{\xi }}\leftarrow \xi_{t}-\iota g_{l}$, combining formula (5), the final cloud-edge-end collaborative system data aggregation global model $\overset{l}{\underset{t+1}{\xi }}$ is obtained as follows:


$$\xi_{t+1}\leftarrow \sum_{l=1}^{L}\frac{q_{l}}{q}\overset{l}{\underset{t+1}{\xi }}$$ (6)

This model includes all data from all terminal devices, thereby completing the terminal data aggregation for the cloud-edge-end collaborative system, facilitating subsequent intelligent data caching.

This study adopts a federated learning framework, whose core advantage is that terminal data does not need to leave the local device, only uploading model updates (such as gradients or parameters), thus avoiding direct leakage of raw sensitive information at the data level and providing a basic guarantee for privacy protection. At the practical application level, this mechanism can effectively support edge collaborative processing of sensitive information such as medical data and user behavior records, complete global model training and caching decisions without centralized collection of raw data, and significantly reduce the risk of data leakage during transmission and storage. However, the framework still faces potential privacy risks, such as malicious attackers or untrusted aggregation servers analyzing multiple rounds of uploaded model updates, using methods such as model inversion attacks or member inference attacks to infer specific user training data features or member information. This is particularly noteworthy in high-value data scenarios such as financial identity authentication and personal health records. In addition, the federated averaging algorithm used in this study did not provide strict theoretical privacy guarantees for such inference attacks, and the high-frequency transmission brought by the incremental mechanism also expanded the potential attack surface to some extent. In order to further enhance the privacy robustness of the system, future work may consider integrating privacy enhancement techniques such as differential privacy or secure aggregation. For example, differential privacy mechanisms can be introduced during the local training phase of the terminal to blur model updates by adding controllable noise, thereby providing a quantifiable level of privacy protection at the cost of sacrificing certain model accuracy. Alternatively, secure multi-party computation based aggregation protocols can be used to ensure that model aggregation can be completed in the cloud without decrypting the updated content. These mechanisms, combined with the incremental update architecture proposed in this article, will be a key research direction for achieving high-performance and high privacy coexisting cloud edge collaborative systems.

### 3.2. Data update based on incremental learning

Since the data in terminal devices is dynamically added and continuously evolving, the traditional federated learning training paradigm designed for static datasets cannot effectively adapt. To address this, this chapter proposes a two-stage adaptive incremental federated update mechanism [10]. This mechanism integrates online incremental learning with lightweight replay principles, enabling efficient learning of dynamic data streams and timely model updates within the federated learning framework. Specifically, we employ an incremental fine-tuning strategy to locally update models with newly added data. Through a Mahalanobis distance-based device selection mechanism and an adaptive weighted aggregation strategy based on influence factors, we achieve the absorption of new knowledge and stable global model updates under federated constraints that prohibit storing historical raw data.

Before each round of updates to the data aggregation global model, the terminal devices participating in the data update must first be determined based on their terminal device level values. The level values can be calculated using the Mahalanobis distance [11] between the current terminal device dataset and the previous terminal device dataset.

The Mahalanobis distance between data primarily reflects the covariance distance between data. Based on the Mahalanobis distance, the similarity between two data points or datasets can be effectively obtained [12,13]. Applying the Mahalanobis distance to the performance metrics of terminal datasets in a cloud-edge-end collaborative system reflects the update volume of the terminal device’s data by measuring the Mahalanobis distance between the current dataset and the previous dataset’s performance metrics within the terminal device. The smaller the Mahalanobis distance, the greater the similarity between the performance metrics of the current dataset and the previous dataset in the terminal device, indicating that the data update volume of the terminal device during this time period is relatively small. Therefore, the terminal device is not included in the current round of data updates. Conversely, the larger the Mahalanobis distance, the smaller the similarity, indicating that the data update volume of the terminal device during this time period is relatively large. The terminal device is included in the current round of data updates. The specific process is as follows:

Based on the accuracy, loss value, and kappa value of the terminal data local aggregation model, construct the feature vectors for the terminal devices. The feature vectors $o_{l}\left(y\right)$ for the l th terminal devices at the current time y and $o_{l}(y-1)$ from the previous update are described as follows:


$$\left\{ \begin{array}{c} @l@o_{l}\left(y\right)=\left(o_{acc}\left(y\right),o_{loss}\left(y\right),o_{kappa}\left(y\right)\right)\\ o_{l}(y-1)=\left(o_{acc}\left(y-1\right),o_{loss}\left(y-1\right),o_{kappa}\left(y-1\right)\right) \end{array}\right.$$ (7)

Among these, $o_{acc}$, $o_{loss}$ and $o_{kappa}$ represent the accuracy, loss value, and kappa value of the local aggregated data model for the terminal, respectively.

To evaluate the stability and trend consistency of data feature changes in terminal devices between consecutive update cycles, this paper introduces covariance as the core metric. This indicator is used to calculate the degree of linear correlation between the current device feature vector and the previous round feature vector. The larger the covariance value, the more consistent the device’s changing behavior in multiple performance dimensions such as accuracy, loss value, and Kappa value, indicating that the device’s data updates have a stable pattern; On the contrary, it indicates that its performance fluctuations are relatively random and its update behavior is not obvious. This covariance measure is not only the basis for subsequent Mahalanobis distance calculations, but also provides a quantitative basis for selecting devices with significant data updates, thus supporting the device selection mechanism in incremental updates. The covariance A between the feature vector $o_{l}\left(y\right)$ of the l th terminal device at the current time y and the feature vector $o_{l}(y-1)$ from the previous update is described as follows:


$$A=\sum_{y(y-1)}Cov\left(o_{l}\left(y\right),o_{l}\left(y-1\right)\right)=R\left[\left(o_{l}\left(y)-\nu_{l}(y\right)\right)\left(o_{l}\left(y-1)-\nu_{l}(y-1\right)\right)\right]$$ (8)

Where, R denotes the mathematical expectation; $\nu_{l}\left(y\right)=R\left(o_{l}\left(y\right)\right)$; $\nu_{l}(y-1)=R\left(o_{l}\left(y-1\right)\right)$; $R\left(o_{l}\left(y\right)\right)$ and $R\left(o_{l}\left(y-1\right)\right)$ represent the mean values of $o_{l}\left(y\right)$ and $o_{l}(y-1)$, respectively.

The Mahalanobis distance $Q\left(\nu_{l}\left(y\right),\nu_{l}\left(y-1\right)\right)$ between $o_{l}\left(y\right)$ and $o_{l}(y-1)$ is described as follows:


$$Q\left(\nu_{l}\left(y\right),\nu_{l}\left(y-1\right)\right)=\sqrt{{\left(\nu_{l}(y)-\nu_{l}\left(y-1\right)\right)}^{Y}A^{-1}\left(\nu_{l}\left(y\right)-\nu_{i}(y-1)\right)}$$ (9)

Set the threshold α, and include all terminals $Q\left(\nu_{i}\left(y\right),\nu_{i}\left(y-1\right)\right)>\alpha$ in this round’s data update.

Select any one terminal z from all terminals requiring updates in this round. Assume that the proportion of new data in terminal z relative to all data in z is $E_{z}$. The specific description of $E_{z}$ is as follows:


$$E_{z}=\frac{\left|O_{z}\right|}{\left|G_{z}\right|}$$ (10)

Where, $O_{z}$ and $G_{z}$ represent the newly added data volume and the total data volume of the terminal device at the current moment, respectively.

Given that at time y−1, the impact of newly added data in the terminal device z on the data aggregation global model is ${℘}_{y-1,z}$, then the impact ${℘}_{y,z}$ of newly added data in z on the data aggregation global model is defined as follows:


$${℘}_{y,z}=E_{z}{℘}_{y-1,z}$$ (11)

Influence reflects the impact of newly added data on the aggregation performance of the global model during the process of data aggregation across all terminal devices [14]. This value can to some extent reflect the value of the terminal dataset. Based on this characteristic, terminal devices with higher influence ${℘}_{y,z}$ should be assigned higher aggregation weights and have their increase rates set to lower levels. To further balance the absorption of new knowledge and model stability in incremental updates, it is necessary to adjust the weights based on the influence of different devices. To achieve this, an incremental decay function based on the hyperbolic tangent function is introduced. The design of this function is inspired by importance weighting methods in continual learning theory (such as elastic weight consolidation). By progressively saturating the weights for high-influence devices, it enhances the retention of critical updates; while simultaneously attenuating low-influence updates to mitigate interference from irrelevant or noisy data on the global model. This approach theoretically aligns more closely with the stability-plasticity balance principle in incremental learning. Therefore, a hyperbolic tangent function is used to establish the incremental decay function $\vartheta_{y,z}$ for terminal devices z. The specific description of $\vartheta_{y,z}$ is as follows:


$$\vartheta_{y,z}={℘}_{y,z}tanh\left(\vartheta_{y-1,z}\right)$$ (12)

This function maps the influence degree to the (0,1) interval and has the characteristics of monotonically increasing and smoothly bounded. It is suitable for adaptive adjustment of device update weights in federated incremental scenarios. The theoretical basis is that the tanh function approximately grows linearly when the influence is low, which is beneficial for initial learning; When the impact is high, it tends to saturate, avoiding excessive dominance of a single device in the aggregation process, which meets the requirements of fairness and robustness in federated learning.

The influence of terminal data on the aggregation model determines the contribution of the terminal data model to the entire data aggregation process [15], distinguishing the utilization value of different terminal data models and obtaining the optimal solution for the deviation issues caused by new data. Therefore, based on the incremental decay function $\vartheta_{y,z}$ of terminal device z, the global aggregation weighting formula for the new data edge of the d th terminal is as follows:


$$\overset{\text{'}}{\underset{r,d}{\xi }}=\sum_{z=1}^{X}\frac{\overset{\text{'}}{\underset{z}{m}}}{m\text{'}}\vartheta_{y,z}\overset{\text{'}}{\underset{y,z}{\xi }}$$ (13)

Where, $\overset{\text{'}}{\underset{r,d}{\xi }}$ represents the global aggregation model for new data at the d th edge device; $\overset{\text{'}}{\underset{y,z}{\xi }}$ represents the local data aggregation model for terminal z in the cloud-edge-end collaborative system; $\overset{\text{'}}{\underset{z}{m}}$ represents the total amount of new data at terminal z; and m' represents the total amount of new data across all terminal devices in the cloud-edge-end collaborative system.

Upload the global aggregation model of newly added data from all edge devices in the cloud-edge-end collaborative system to the cloud to obtain the final global aggregation model of newly added data $\overset{\text{'}}{\underset{y}{\xi }}$. The description of $\overset{\text{'}}{\underset{y}{\xi }}$ is as follows:


$$\overset{\text{'}}{\underset{y}{\xi }}=\sum_{d=1}^{D}\frac{\overset{\text{'}}{\underset{d}{m}}}{M\text{'}}\overset{\text{'}}{\underset{r,d}{\xi }}$$ (14)

Among these, $\overset{\text{'}}{\underset{d}{m}}$ represents the total amount of new data added to the edge of the cloud-edge-end collaborative system in the d th edge; M' represents the total amount of new data added to the cloud of the cloud-edge-end collaborative system.

By combining the global aggregation model of newly added data at the time y and the corresponding original data global aggregation model $\xi_{y+1}$ from Section 2.1, we obtain the weighted aggregated global model $\xi_{y}$ after data updates. The description of $\xi_{y}$ is as follows:


$$\xi_{y}\leftarrow \frac{M\xi_{y+1}+M\text{'}\overset{\text{'}}{\underset{y}{\xi }}}{M+M\text{'}}$$ (15)

Where, M represents the total data volume in the original global aggregation model, i.e., the total data volume of all terminals before data update. Based on the weighted aggregation global model after data update $\xi_{y}$, real-time update aggregation of terminal data can be achieved [16], and $\xi_{y}$ includes the new data and original data from the cloud-edge-end collaborative system terminals.

In cloud edge collaborative intelligent caching, the update aggregation effect of terminal data directly affects the final data caching result. The better the data update aggregation effect, the higher the hit rate of cached data, that is, the higher the data caching performance. The cloud edge collaborative intelligent caching method based on incremental federated learning algorithm is now used to update the data in terminal devices, and aggregate the data after each update. The aggregation effect of the first 15 updates can be judged by the matching degree between the aggregated data and the newly added raw data in the terminal. This article defines matching degree as the degree of consistency between the weighted aggregated global model and the locally added data on the terminal in predicting popularity. Specifically, after each incremental update, the predictive performance of the global model aggregated in the cloud is tested based on the locally added data of the terminal device and its true popularity label. The matching degree is measured by calculating the popularity classification accuracy of the global model for newly added data on the terminal. The calculation formula is as follows:


$$M=\frac{\text{1}}{N}\sum_{k=1}^{N}\left(\frac{\text{1}}{\left|\overset{k}{\underset{new}{D}}\right|}\sum_{j=1}^{\left|\overset{k}{\underset{new}{D}}\right|}I(G_{t\;}\left(x_{j\;}\right)=y_{j}\;)\right)$$ (16)

Among them, N represents the number of terminal devices participating in the update in this round; $\left|\overset{k}{\underset{new}{D}}\right|$ represents the newly added data volume of terminal k; $G_{t\;}\left(x_{j\;}\right)$ represents the predicted label of the global model for sample $x_{j\;}$; $y_{j\;}$ is the true popularity label of sample $x_{j\;}$; I(·) represents the indicator function. If the prediction is correct, it is 1; otherwise, it is 0.

**Fig 2:**
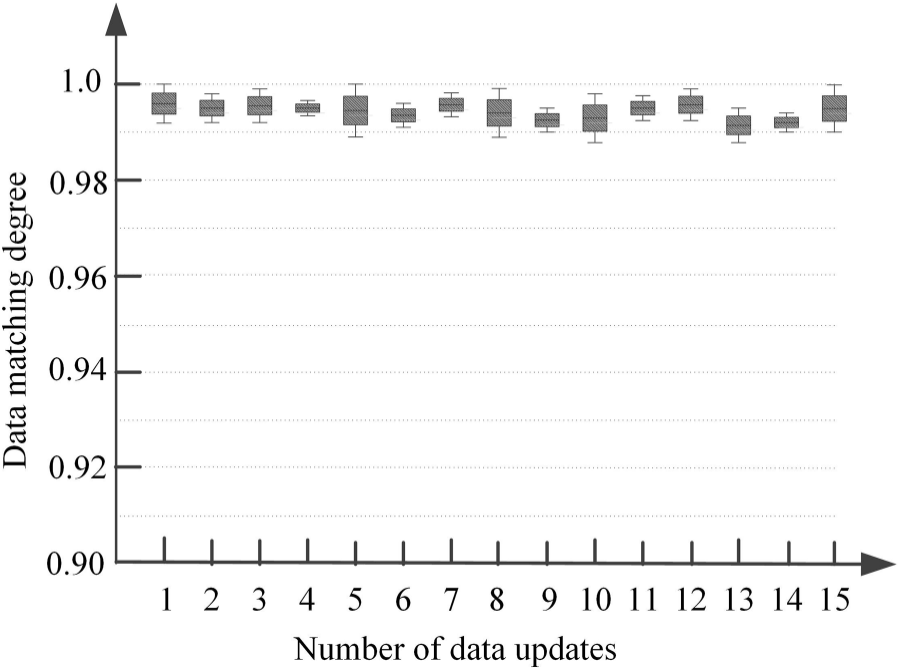
Data Matching Degree.

This matching degree measures the capability of the aggregated global model. The capability is to absorb and reflect newly added data features from each terminal. A higher matching degree indicates stronger consistency. The consistency is between the aggregated new data and the original new data on the terminal. Stronger consistency means better data update aggregation performance. Fig 2 shows this matching degree.

As shown in Fig 2, the upper and lower boundary lines of the box represent the maximum and minimum matching degrees between aggregated data and original data in a single update, respectively. The median line indicates the average matching degree of the data group in a single update, while the length of the box reflects the fluctuation range of the matching degree. For each update, the matching degree between the aggregated results and the newly added data in the terminal is higher than 0.98, with the maximum matching degree reaching 1.0. Additionally, the fluctuation range of the matching degree after each update is small, indicating that the incremental federated learning algorithm proposed in this method can effectively update and aggregate terminal data, with high aggregation stability, providing a reliable foundation for subsequent data caching.

## 4. Cloud-edge-end data collaborative intelligent caching strategy

For data in the cloud-side weighted aggregation global model, the caching order is first determined by data popularity; Second, considering the actual capabilities of the nodes, the weight values and cache spaces of each edge and end node are calculated, and the data is cached in order of popularity from high to low in nodes with weight values from high to low. When the cache space of the node with the highest weight value is full, the data is cached in the node with the next highest weight value; Finally, when all edge nodes have no cache space, data cache replacement is completed based on the importance of the new data and the importance of the already cached data in the nodes.

### 4.1. Data cache order acquisition based on popularity

In a cloud-edge-end collaborative system, each edge node has a limited amount of content, and the popularity of data within a single node no longer follows the Zipf distribution [17]. Therefore, it is necessary to identify more appropriate parameters to calculate the popularity of data within nodes, ensuring that data retains maximum value within nodes while maintaining overall network performance and improving the hit rate of user requests within edge nodes. The popularity calculation model needs to balance the freshness and popularity of data, in order to maximize the value of cached data within nodes and improve the overall network request hit rate.

Comprehensively considering the number of times data is accessed across edge nodes and the most recent access time, we accurately predict data popularity [18], where $U_{i}$ denotes the current time of data i, $U_{i-recent}$ denotes the most recent access time of data i, and $U_{i-first}$ denotes the first access time of data i within the edge node. The time interval $U_{i-int}$ between the current time and the most recent access time is defined as follows:


$$U_{i-int}=U_{i}-U_{i-recent}$$ (17)

The smaller the time interval, the fresher the data and the higher the likelihood of it being accessed again.

To eliminate the dimensional differences in time intervals between different data and ensure that their values fall within a unified and comparable range (such as [0,1]), standardization is required. Define $U_{i-int-max}$ as the maximum time interval corresponding to $U_{i-int}$ among all data. Then, the normalized distance $U_{i-int-normal}$ to the last access time is defined as follows:


$$U_{i-int-normal}=\frac{U_{i-int}}{U_{i-int-max}}$$ (18)

The average access interval $U_{i-aver}$ of the data i can be expressed as:


$$U_{i-aver}=\frac{U_{i-recent}-U_{i-first}}{W_{i}}$$ (19)

Where $W_{i}$ represents the number of times $U_{i-int}$ was requested. Assuming that $U_{aver-max}$ represents the maximum value of $U_{i-aver}$ across all data, normalize $U_{i-aver}$ to obtain $U_{i-aver-normal}$ as follows:


$$U_{i-aver-normal}=\frac{U_{i-aver}}{U_{aver-max}}$$ (20)

Similarly, normalizing the average access interval aims to eliminate the magnitude impact of different data access frequencies and convert it into comparable values within the range of [0,1].

The popularity $S_{i}$ of data i in the global model of cloud data aggregation is as described below:


$$S_{i}=\frac{1/(U_{i-int-normal}+1)}{U_{i-aver-normal}}=\frac{1/((U_{i}-U_{i-recent})/U_{int-max}+1)}{(U_{i-recent}-U_{i-first})/W_{i}/U_{aver-max}}$$ (21)

The smaller the $U_{i-int}$ value, the shorter the time interval since the data was last accessed, indicating higher freshness and a greater probability of being accessed; The smaller the $U_{i-aver}$ value, the more times the data was accessed within a short period, indicating that the data is in a high-demand phase and is being requested by more users. The larger the $S_{i}$ value, the higher the freshness of the data i, and the higher the popularity, meaning that the data i can effectively meet the requests of subsequent users.

Calculate the popularity of each data point in the cloud data aggregation global model using formula (20), sort the data points by popularity from highest to lowest, and cache them sequentially on edge nodes based on the sorting order.

The above symbol table is shown in Table 1.

**Table 1:** Symbol Table.

symbol	definition	Domain/Unit Definition
$U_{i}$	the current time of data i	timestamp
$U_{i-recent}$	the most recent access time of data i	timestamp
$U_{i-first}$	the first access time of data i within the edge node	timestamp
$U_{i-int}$	The time intervalbetween the current time and the most recent access time	s
$U_{i-int-max}$	the maximum time interval corresponding to $U_{i-int}$ among all data	s
$U_{i-int-normal}$	the normalized distance to the last access time	[0,1]
$U_{i-aver}$	The average access interval of the data i	s
$W_{i}$	represents the number of times $U_{i-int}$ was requested	frequency
$U_{aver-max}$	the maximum value of $U_{i-aver}$ across all data	s
$U_{i-aver-normal}$	ormalize $U_{i-aver}$	[0,1]

### 4.2. Determining data caching locations based on node weight and cache space

The configuration of cache space sizes for edge nodes significantly impacts the performance of cloud-edge-end systems. If the cache space sizes for edge nodes in the cloud-edge-end collaborative system are arbitrarily configured, this may lead to inefficiencies, such as cache space waste in regions with low user demand or insufficient cache space in regions with high user demand. To improve the performance of the cloud-edge-end collaborative system and reduce economic investment, it is necessary to configure appropriate cache space sizes for each edge node.

The cloud – edge – end collaborative intelligent caching method based on the incremental federated learning algorithm comprehensively considers user demands and cloud-edge-end network topology information to design a cache size allocation scheme for edge – end nodes, and it is a heterogeneous cache space allocation scheme [19]. The cloud – edge – end collaborative intelligent caching method based on the incremental federated learning algorithm classifies the data in the global model of cloud – side data aggregation into T types. Through demand research, the demand quantity $\mu_{ix},x\in T$ of the i th terminal user for various types of data per unit time is obtained. Then, the total demand quantity $w_{x}$ of all terminal users for the x th type of data per unit time can be obtained. The specific description of $w_{x}$ is as follows:


$$w_{x}=\sum_{i\in P}\mu_{ix}$$ (22)

In the formula, P represents the set of end-user clients in the cloud-edge-end collaborative system.

Assuming the average size of data of type x is $h_{x}$, the size of $h_{x}$ is obtained by taking the average of the sizes of similar data in the actual cloud-edge-end collaborative system. The total size $V_{y}$ of the data cache space required for the entire cloud-edge-end collaborative system is calculated as follows:


$$V_{y}\text{=}\sum_{x=1}^{T}(w_{x}\times h_{x})$$ (23)

Once the total size of the data cache required for all cloud-edge-end collaborative systems is determined, it is allocated to the various edge nodes according to specific rules. The Cloud-Edge-End Collaborative Intelligent Caching Method Based on Incremental Federated Learning Algorithm Design allocation rules based on the cloud-edge-end network topology and user requirements. When considering topology information factors, select the following metrics for the cloud-edge-end network topology:

(1) Node degree weight $e_{i}$. $e_{i}$ is related to the degree of the edge – end node $b_{i}$ and the degrees of all edge – end nodes in the cloud – edge – end network area. The degree of an edge – end node refers to the number of links associated with the edge – end node. For a directed graph, the in – degree of an edge – end node refers to the number of links entering the edge – end node, and the out – degree refers to the number of links starting from the edge – end node. The cloud – edge – end collaborative intelligent caching method based on the incremental federated learning algorithm is for undirected graphs, so the in – degree and out – degree are not distinguished. The formula for calculating the degree weight $e_{i}$ is as follows:


$$e_{i}=k_{i}/\sum_{j\in B}k_{j}$$ (24)

In the formula, $k_{i}$ represents the degree of the edge node $b_{i}$; B represents the set of edge nodes of the edge node in the cloud-edge-end collaborative system; $\sum_{j\in B}k_{j}$ represents the sum of the degrees of all edge nodes in the cloud-edge-end collaborative system.

(2) The edge-end node density $v_{i}$. $v_{i}$ reflects the distance relationship between the edge-end node $b_{i}$ in the edge-cloud-end collaboration system and the remaining edge-end nodes in the system. The formula for calculating $v_{i}$ is:


$$v_{i}=1/\sum_{j\in B}t\left(i,j\right)$$ (25)

Where, t(i,j) is the shortest path hop count from the edge node $b_{i}$ to the edge node $b_{j}$.

(3) The centrality $p_{i}$ of the edge node. $p_{i}$ involves the distance from the edge node $b_{i}$ to the farthest node, and the formula for calculating $p_{i}$ is as follows:


$$p_{i}=1/\underset{j\in B}{max}t\left(i,j\right)$$ (26)

Where, $\underset{j\in B}{max}t\left(i,j\right)$ is the maximum hop count in the shortest path from edge node $b_{i}$ to all edge nodes.

When considering user demand factors, the based on the incremental federated learning algorithm in the cloud-edge-end collaboration intelligent caching method uses the request impact metric, which simultaneously reflects user demand and edge node importance. Assume that $I_{i}$ represents the total number of requests transmitted from other edge nodes to the edge node $b_{i}$ in the cloud-edge-end collaborative system. The cloud-edge-end collaborative intelligent caching method based on the incremental federated learning algorithm uses the total number of requests as the metric for request influence, obtaining the request influence $I_{i}$ of the edge node $b_{i}$ as follows:


$$I_{i}=\sum_{i\in Ba(i\to j)\in C(i\to j)}\sum (\overset{a(i\to j)}{\underset{ij}{x}}\times \prod_{s\in a(i\to j)}(1-serv_{s}{)}^{-1})$$ (27)

Among these, B represents the set of edge nodes in the cloud-edge-end collaborative system; a(i→j) is a random path from the edge node $b_{i}$ to the edge node $b_{j}$; C(i→j) is the set of all random paths from the edge node $b_{i}$ to the edge node $b_{j}$; s denotes the nodes on the random path a(i→j) from the edge node $b_{i}$ to the edge node $b_{j}$; $serv_{s}$ represents the service rate of node s, i.e., the probability that a data request is successfully processed at edge node $b_{i}$ or found in the pending request table (PIT) with the same entry; $\overset{a(i\to j)}{\underset{ij}{x}}$ represents the total number of various data requests transmitted from edge node $b_{i}$ along path a(i→j) to edge node $b_{j}$ within a unit time.

Considering the four metric indicators of the edge-end node degree weight $e_{i}$, compactness $v_{i}$, centrality $p_{i}$, and request influence $I_{i}$ in the cloud-edge-end collaborative system [20], the metric indicators are weighted, and the weighted value N(i) of the edge-end nodes $b_{i}$ is obtained. The weighted expression for N(i) is:


$$N\left(i\right)=\beta \times e_{i}+\chi \times v_{i}+\delta \times p_{i}+\varepsilon \times I_{i}$$ (28)

Where, β, χ, δ and ε represent the weight coefficients of the metric indicators $e_{i}$, $v_{i}$, $p_{i}$ and $I_{i}$, respectively.

The cache space size $V\left(b_{i}\right)=$ configured for the edge nodes $b_{i}$ is described as follows:


$$V\left(b_{i}\right)=V_{y}\times \frac{N\left(i\right)}{\sum_{j\in B}N\left(j\right)}$$ (29)

In summary, after obtaining the weight values [21] of each edge node in the cloud-edge-end collaborative system, sort the nodes in descending order of weight values. The edge nodes cache the data in the edge node with the highest weight value in descending order of popularity. Calculate the cache space size for each node using formula (28). When the amount of data cached in the node with the highest weight value reaches its cache space size, the remaining uncached data is cached in the edge nodes with the next highest weight values in descending order of popularity, and the data aggregation is completed for all data in the global model.

### 4.3 Cache replacement based on data importance

Due to the continuous update of data in the cloud-edge-end collaborative system, the amount of data that needs to be cached in edge-end nodes is constantly increasing. When the available cache space of each edge-end node is 0, cache replacement for new data is required [22]. The principle of cache replacement is as follows: each time data in the cache space needs to be replaced, the data with the smallest importance is replaced first; if the cache space is still insufficient, the data with the second smallest importance is replaced, and this process continues until the new data can be stored in the edge-end node. If the importance of the current new data is lower than that of the data with the smallest importance in the cache space of the edge-end node, no replacement of the existing data in the cache space will be performed, and the new data will not be cached at this time.

In the cloud-edge-end collaborative intelligent caching method based on the incremental federated learning algorithm, the importance [23] of data is calculated based on the customer request level of the data at the edge-end node and the request frequency of the data in corresponding different time periods. For the newly added data f, the description of its importance $H_{f}$ at the current edge-end node is as follows:


$$H_{f}=\sum_{r=min\left\{r_{f}\right\}}^{max\left\{r_{f}\right\}}\psi_{f_{r_{a}}}$$ (30)

Among them, $r_{a}$ represents the average request level of terminal users for the newly added data f; $min\left\{r_{f}\right\}$ and $max\left\{r_{f}\right\}$ respectively represent the minimum request level and the maximum request level of the newly added data f at each edge – end node in the cloud – edge – end system; $\psi_{f_{r_{a}}}$ represents the weight value of the newly added data f when its request level is $r_{a}$. For the average request level $r_{a}$ of terminal users for the newly added data f and the weight value $\psi_{f_{r_{a}}}$ when its request level is $r_{a}$, the calculation process is shown in formula (30):


$$\left\{ \begin{array}{c} @l@r_{a}=\frac{\sum_{r=min\left\{r_{f}\right\}}^{max\left\{r_{f}\right\}}r_{f}g_{fr_{a}}}{J_{yi}}\\ \psi_{f_{r_{a}}}=\frac{r_{a}}{\sum_{r=min\left\{r_{f}\right\}}^{max\left\{r_{f}\right\}}r}\left[\rho_{\text{1}}\sum_{y-\Delta y}^{y}G_{ir}\left(y\right)+\rho_{\text{2}}\sum_{y-2\Delta y}^{y-\Delta y}G_{ir}\left(y\right)+\rho_{\text{3}}\sum_{y-3\Delta y}^{y-2\Delta y}G_{ir}\left(y\right)\right] \end{array}\right.$$ (31)

Where, $r_{f}$ represents the request level of the newly added data f; $g_{fr_{a}}$ represents the request frequency of the newly added data f at the request level $r_{f}$; $J_{yi}$ represents the total request frequency of the newly added data f at the current node; $\sum_{r=min\left\{r_{f}\right\}}^{max\left\{r_{f}\right\}}r_{f}g_{fr_{a}}$ represents the total product of the request frequency of all users sending the newly added data f at the current node and the corresponding request levels; y represents the current time; Δy represents the time period; $G_{ir}\left(y\right)$ represents the request frequency of the newly added data f at the request level r within a certain time period; $\rho_{i}(i\in \left\{1,2,3\right\})$ represents the proportion of requests for newly added data f during different time periods, A cloud-edge-end collaborative intelligent caching method based on incremental federated learning algorithm Take data from three time periods (larger time periods can be obtained by adjusting Δy), i.e., $\rho_{\text{1}}+\rho_{\text{2}}+\rho_{\text{3}}=1$, with the aim of excluding cases where the client’s request frequency for data from a long time ago is very high and the recent request frequency is very low, resulting in a high overall request frequency.

In summary, by combining the average request level and total frequency of data, the importance $H_{f}$ of data during a certain time period is calculated. Based on the data importance $H_{f}$, data is replaced to better reflect data real-time and provide users with a better access experience while improving network efficiency.

## 5. Experiment and discussion

To validate the effectiveness of the cloud-edge-end collaborative intelligent caching method based on incremental federated learning, testing is required.

The experiment was conducted in the cloud-edge-end collaborative architecture shown in Fig 1, with the following experimental parameters set:

To verify the effectiveness of the proposed method, this experiment constructed a dataset by mixing public and self built data, with a total of 5 × 10 ⁴ request records, divided into training and testing sets in a 4:1 ratio. Data sourced from MovieLens 20M public dataset (https://grouplens.org/datasets/movielens/20m/) And based on this, expand and simulate: extract its user ID, content ID, timestamp, and rating (mapped to request priority) as the real behavior benchmark; At the same time, in order to adapt to cloud edge end caching scenarios, six types of content were generated through script supplementation, including videos, documents, images, audio, applications, and web pages. The request distribution of the above content follows Zipf’s law (shape parameter α=0.8) to simulate the heat difference in real scenes; The data size of each content is uniformly randomly generated within the range of 10 KB to 500 KB; Each request record is associated with a randomly assigned source terminal number. Based on the quartiles of historical access frequency, label each content with popularity tags (divided into high, medium, and low levels). All user identifiers and content identifiers are anonymized, covering a continuous 7-day time series to simulate a dynamically changing request flow; The cloud edge system consists of one cloud, four edge devices, and 200 terminal devices, with each edge managing 50 terminal devices. Each device is randomly assigned 1000 training data and 1000 testing data; The aggregation frequency of edge to terminal data is 50 times, and the aggregation frequency of edge to terminal data in the cloud is 10 times. The local data aggregation model on each terminal device adopts a three-layer fully connected neural network, consisting of an input layer, two hidden layers (containing 64 and 32 neurons respectively), and an output layer, with an activation function of ReLU. Federated learning aggregation uses the FedAvg algorithm, with a local learning rate of 0.01 and a batch size of 32. Each terminal undergoes 5 local training rounds in each round of communication. The convergence condition of the global model is set to a continuous two round loss value change of less than 0.001 or reaching a maximum communication round of 50. In incremental updates, the Mahalanobis distance threshold is set to 0.15 to determine whether the terminal participates in this round of updates; When triggering an update, the batch size is 16, the initial learning rate is 0.005, and a decay strategy is adopted; the data is divided into 6 categories; the weight coefficients for the node weight measurement indicators are set to β=χ=δ=0.3, and ε=0.1; the cache size for each edge node is 70MB. All experiments were run on the same server with a hardware configuration of Intel Xeon Gold 6248 CPU (2.5 GHz, 20 cores), 64 GB RAM, and NVIDIA RTX 3090 GPU (24 GB of graphics memory). The operating system is Ubuntu 20.04, and the deep learning framework is PyTorch 1.12.0.

To validate the rationality of the weight coefficient settings in Equation (27), a set of comparative experiments was designed. By varying the combinations of the four weight coefficients (β,χ,δ,ε), their impact on system cache balance and hit rate was evaluated. The experiments were conducted under the same dataset and network topology, with all other parameters fixed and only the weight allocation adjusted. The value range for each weight coefficient was set to [0.1, 0.4], satisfying the constraint β+χ+δ+ε=1. Table 2 presents seven representative weight combinations and their corresponding performance metrics.

**Table 2:** Cache Performance Comparison under Different Weight Coefficient Combinations.

Exp. Group	Weight Coefficients (β,χ,δ,ε)	Cache Balance	Cache Hit Rate
1	(0.4, 0.3, 0.2, 0.1)	0.872	0.831
2	(0.3, 0.4, 0.2, 0.1)	0.885	0.829
3	(0.3, 0.3, 0.3, 0.1)	0.901	0.846
4	(0.2, 0.3, 0.3, 0.2)	0.879	0.837
5	(0.25, 0.25, 0.25, 0.25)	0.864	0.822
6	(0.35, 0.25, 0.25, 0.15)	0.887	0.835
7	(0.3, 0.2, 0.4, 0.1)	0.894	0.838

As shown in Table 2, when the weight allocation is biased towards a single metric (e.g., Groups 1, 2, and 7), the system performance remains at a certain level but does not reach the optimum. In Group 4, increasing the weight for request influence leads to a slight decline in both balance and hit rate. Group 5 employs a uniform allocation strategy (0.25 each), resulting in average but not outstanding performance. Although Group 6 is close to optimal, it is slightly inferior to Group 3. The weight combination adopted in this paper (Group 3) achieves the highest values in both cache balance and hit rate, at 0.901 and 0.846, respectively. This indicates that this weight allocation can more comprehensively coordinate node topological structure and user request characteristics, thereby achieving superior collaborative caching performance. Furthermore, this combination assigns relatively balanced importance to the four metrics (with request influence being slightly lower), aligning with the design principle of giving equal consideration to both node capability and user demand in cloud-edge-end collaborative systems, demonstrating strong rationality and practicality. It should be noted that the optimal value is obtained through experimental comparison based on a specific experimental scenario in this article (including dataset, network topology, request distribution, etc.). The selected weight coefficients (0.3, 0.3, 0.3, 0.1) depend on the current experimental configuration and are not a universal optimal solution applicable to all cloud edge systems.

**Fig 3:**
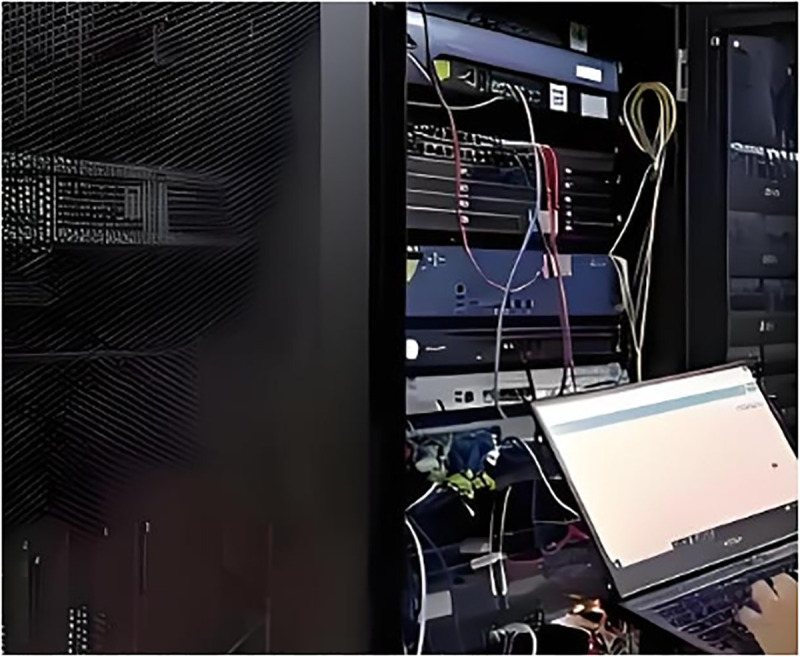
Cloud-Edge-End Collaborative Intelligent Caching Experimental Testing Scenario.

The experimental test scenario is shown in Fig 3.

(1) Cache Balancing Degree

**Fig 4:**
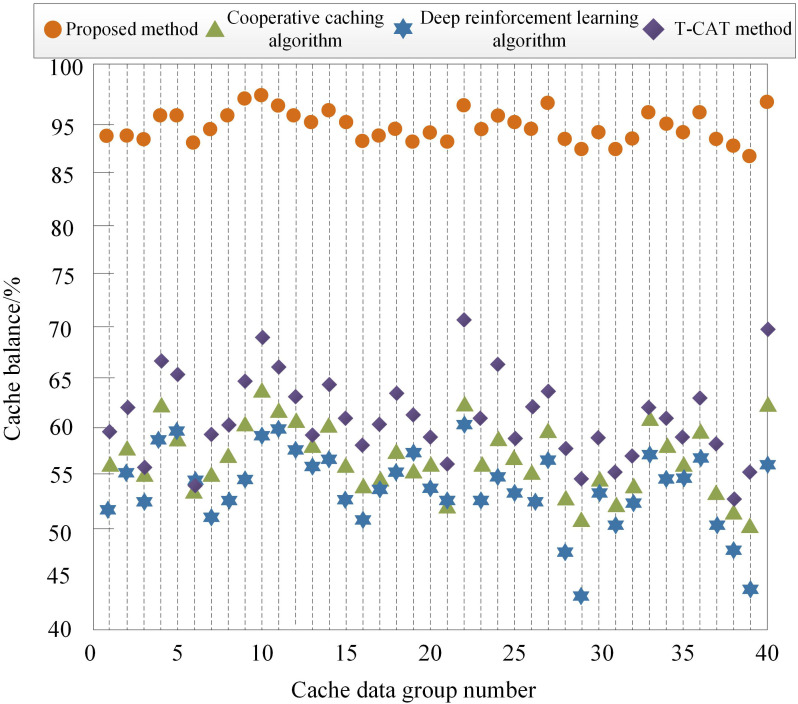
Cache Balance.

When the data cache balance in a cloud-edge-end collaborative system is poor, it can lead to a decline in overall system performance. Therefore, the higher the data cache balance, the more beneficial it is for improving overall system performance, and it also indicates that the data caching method has higher performance. We now apply a cloud-edge-end collaborative intelligent caching method based on incremental federated learning algorithms, cooperative caching algorithms, deep reinforcement learning algorithms, and the T-CAT method to cache data groups with varying data volumes. The cache balancing of these groups is shown in Fig 4. Among them, this article chooses deep reinforcement learning as the baseline for comparison, mainly based on the following considerations: firstly, deep reinforcement learning is widely used in dynamic caching decision-making and is a representative method in the field of edge intelligent caching; Secondly, its centralized learning paradigm contrasts sharply with the distributed incremental federated architecture proposed in this paper, highlighting its advantages in privacy protection, dynamic adaptability, and collaborative efficiency; Finally, by comparing it in the same scenario, the improvement effect of the proposed method on key indicators such as cache hit rate and balance can be objectively evaluated.

Analysis of Fig 4 shows that the 22nd and 40th groups contain the largest amounts of data, resulting in poorer cache balancing for these two groups across all four methods. However, the proposed method consistently achieves higher balancing than the comparison methods. Due to various disturbances such as noise factors, outliers, and environmental factors, the cache balancing of different methods varies. For the same group of data, the proposed method achieves significantly higher cache balance than cooperative caching algorithms, deep reinforcement learning algorithms, and the T-CAT method. This is primarily because the proposed method comprehensively considers data popularity and node weights during the cache expansion process, effectively caching popular data on nodes with higher weights, thereby ensuring a balanced distribution of cached data.

(2) Cache Hit Rate

Cache hit rate is a key metric for evaluating cloud-edge-end collaborative intelligent caching. It reflects the ratio of the number of times cached data is accessed to the total number of user requests to the system. A higher cache hit rate indicates a higher probability of successfully accessing cached data and better data caching performance. The calculation process for cache hit rate J is as follows:


$$J=\frac{t}{T}$$ (32)

**Fig 5:**
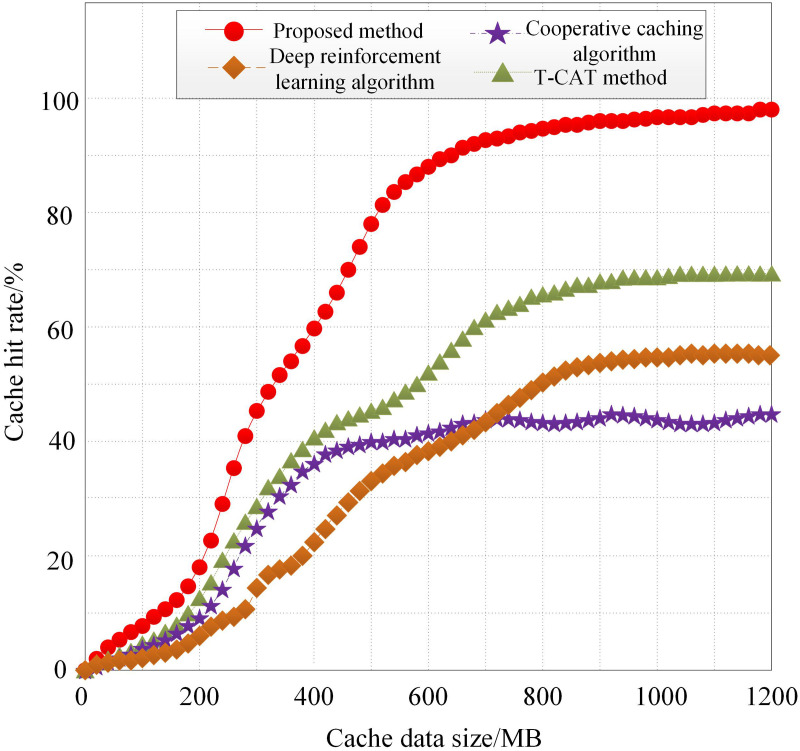
Cache Hit Rate.

Where, t represents the number of cache hits, and T represents the total number of user accesses to the cloud-edge-end collaborative system data. The cache hit rate for the cloud-edge-end collaborative intelligent caching method based on incremental federated learning, the cooperative caching algorithm, the deep reinforcement learning algorithm, and the T-CAT method is calculated. The changes in the cache hit rate with varying cache data sizes are shown in Fig 5.

As shown in Fig 5, as the cache data size increases, the cache hit rates of all four methods gradually increase, and the growth rates of the cache hit rates all exhibit a “fast-then-slow” characteristic. This is because, in the initial stage of cache data, the types of cache data increase, and customer access demands are gradually met, leading to a rapid increase in the cache hit rate. However, as the cache data continues to grow, the types of cache data reach saturation, and duplicate data within the cache increases, resulting in a slower increase in the cache hit rate. Compared to the cooperative caching algorithm, deep reinforcement learning algorithm, and T-CAT method, the proposed method demonstrates significantly higher cache hit rates and faster growth rates. This indicates that the proposed method can prioritize caching data that meets user access requirements, thereby reducing response times. In other words, the proposed method achieves better data caching performance and higher overall system performance.

## 6. Conclusion

This article proposes an intelligent caching method based on incremental federated learning algorithm to address the issues of insufficient data privacy protection, poor dynamic adaptability, and uneven allocation of cache resources in cloud edge collaborative environments. By designing a two-stage adaptive aggregation mechanism and introducing an incremental update strategy in the federated learning framework, dynamic aggregation and privacy protection of terminal data have been achieved; The differentiated cache placement strategy combining data popularity and node weights has improved cache balance; And based on data importance, cache replacement is implemented to enhance adaptability to dynamic requests. The experimental results show that the proposed method performs well in key indicators such as data aggregation matching degree, cache balance degree, and hit rate. The matching degree is stable above 0.98, the balance degree reaches 0.901, and the highest hit rate is 0.846, which is significantly better than existing methods. However, there are still certain limitations to this study, including the fact that although the experimental dataset covers multiple types of content, its dynamics and scale still differ from real network environments; The scalability of the system has not been fully validated when the number of nodes increases significantly; The trade-off between communication overhead and privacy protection strength in the process of federated learning needs further optimization. Future work will revolve around the following directions: introducing large-scale dynamic datasets that are closer to real-world scenarios for validation; Explore distributed aggregation mechanisms to support larger scale deployment of edge nodes; Integrating privacy enhancement technologies such as differential privacy or secure multi-party computation to improve system privacy protection capabilities while ensuring model performance.
